# Editorial

**Published:** 1871-03

**Authors:** 


### Editorial.

***Retirement of Professor Blaney.***

James V. C. Blaney, M.D., incumbent of the chair of Chemistry since the organization of Rush Medical College, has resigned the position and has accepted from the Board of Trustees the Emeritus Professorship of the same branch.

Prof. Blaney has been induced to take this course from the large and increasing demands made upon him for his services in the practical application of the science in analyses, assaying, etc., which the state of his health, permanently impaired by his arduous labors and exposures while in the army, precluded his attending to in addition to the onerous duties of his chair.

Whilst we cannot but regret to lose the daily active co-operation of Prof. Blaney as a colleague, we are rejoiced that his unsurpassed practical skill as a chemist is meeting with deserved reward from that portion of the public which needs exquisite skill in this beautiful art.

The Alumni of the College will be pleased to hear that Prof. B., in accepting the Emeritus Professorship, still shows his attachment to the College and his earnest wishes for its continued prosperity.

It is needless to say that wherever he is known he is honored for his varied and profound scholarship, and, better still, is beloved as a whole-souled, high-minded, generous-hearted, courteous and genial gentleman.

***Practical Chemistry.***

Prof. Henry M. Lyman, M.D., who succeeds Prof. Blaney in the chair of Chemistry in Rush Medical College, is now busily reconstructing the laboratory of the building for the purpose of giving practical instruction in his department during the interval between the annual regular sessions. Every appliance for successful prosecution of this important and interesting branch of study will be afforded to students, and facilities which will hereafter render it entirely unnecessary for them to seek other cities, or foreign countries, for perfecting themselves in its practical details.

During the session which has just closed, Prof. Lyman has developed a success in experimentation, a clearness and systematic precision in exposition, a scholarly and thoughtful method, combined with a pleasing style as a lecturer, which have, collectively, surprised all but those sufficiently intimate with him to have become already aware of the strength and vigor he had acquired by long continued, earnest and industrious culture of excellent native powers of mind.

We write these words, not from the merely friendly spirit we naturally feel for a colleague, but because they are eminently merited, and we wish the Alumni of "Rush," and all interested in its welfare to know that we believe the place, so long and honorably filled by Prof. Blaney, is now occupied by a gentleman every way worthy of their confidence and high appreciation.

***Clinics.***

The daily clinics at Rush College are rapidly rising to high importance and interest. The average attendance of patients at the medical clinic, we are informed, ranges from thirty to fifty. The gynecological clinic, on Thursdays, and the surgical, on Saturdays, are proportionately equally well attended.

It is not more than the facts warrant, to say that the facilities for clinical instruction now possessed by the College, and available throughout the entire year, are not only equal to all the wants of students, but unsurpassed in every element of variety, importance and practical advantage by any other similar institution in the large cities of the continent. *En passant*, we must be permitted to say that, in this age of the world, medical colleges elsewhere than in large cities are anachronisms which it would be better for the profession to discountenance, than to seek its elevation by the doubtful agency of protective legislation. What the profession requires for its protection is knowledge—knowledge gained by actual use of the senses, by experiment, by demonstration. The day of dogmatism, however eloquently expounded, is past.

***An Important Discovery.***

During the beginning of the present winter, Dr. R. Dexter, of this city, discovered in his numerous dissections in comparative anatomy, that the spinal cord in the deer, antelope, and some other animals, was mostly if not entirely protected from concussion by an unusual deposit of fatty cellular tissue, instead of aqueous fluid, which was continuous throughout the entire length of the spinal canal and membranes, and not heretofore mentioned. Since his first discovery he has made numerous other dissections, all of which tend to verify his former conclusions. It has stimulated several medical students to work in the same field, hence the necessity of a somewhat premature announcement. This discovery adds another link to the Darwinian chain. *Com.*

We understand that Dr. Dexter has been devoting large attention to comparative anatomy, especially that of the brain and spinal cord. *Palmam qui meruit ferat.* Further confirmation of his observations will be looked for with much interest.

***Chlorate of Potash, etc., in Diarrhœa and Dysentery.***

H. W. Richardson, M.D., of Woodstock, Ill., writes us that during the last season he treated some seventy-five cases of both diarrhœa and dysentery, with rapidly successful results, by the following method:

"No. 1.

℞. Potassæ Chlorat., - - - - ʒj;

Acet. Tinct. Opi., - - - gtt. v—x;

Tinct. Camph., - - - - - ʒss;

Aq., - - - - - - ℥ij;

M. Sig. A teaspoonful every hour or two, as needed, to control the bowels.

To strengthen and improve the digestive organs, and aid digestion:

No. 2.

℞. Baudalt's Pepsin, - - - - ʒss;

Bicarb. Soda, - - - - - ʒj;

Mix by trituration, and add—

Brandy, Water, - - - - ää ℥ss;

Sig. 20 drops in food every three hours.

No. 3. For Diet. When the child gets q. s. healthy mother's milk, don't change—otherwise use—

℞. Beef and Veal, - - - - - ää ℥ij.

Boil four to six hours in a quart of water; add a little salt; skim when cold. Mix this with equal parts of cow's milk and water. 20 drops of pepsin mixture added to one-third teacup of this diet, and given to a child one to three years old, every three hours, will usually digest.

N. B. If patient is feverish, add to No. 1, Tinct. Gelsem. ʒss, or Tinct. Aconite, gtts. v to viij. The discriminating practitioner will understand how to vary the above formula to suit age and circumstances."

***Explanatory.***

A very large proportion of our subscribers are alumni of Rush Medical College, and as such are deeply interested in everything pertaining to it. This must be our apology for devoting so much of our space the present month to its chronicles. It will be seen, however, that very much of this matter is of general interest.

***Back Numbers Wanted.***

To complete files, Twenty-five cents each will be paid for the following Nos. of the JOURNAL for 1869: June 1, No. 11; July 15, No. 14; August 1, No. 15; August 15, No. 16.

